# Consequences of gamma-ray irradiation on structural and electronic properties of PEDOT:PSS polymer in air and vacuum environments†

**DOI:** 10.1039/d1ra03463d

**Published:** 2021-06-10

**Authors:** Aswin kumar Anbalagan, Shivam Gupta, Mayur Chaudhary, Rishi Ranjan Kumar, Yu-Lun Chueh, Nyan-Hwa Tai, Chih-Hao Lee

**Affiliations:** Department of Engineering and System Science, National Tsing Hua University Hsinchu 30013 Taiwan chlee@mx.nthu.edu.tw; Department of Materials Science and Engineering, National Tsing Hua University Hsinchu 30013 Taiwan; Institute of Nuclear Engineering and Science, National Tsing Hua University Hsinchu 30013 Taiwan

## Abstract

In this work, the effects of gamma-ray irradiation (up to 3 kGy) on the structural and electronic properties of poly(3,4-ethylenedioxythiophene):poly(styrenesulfonate) (PEDOT:PSS), irradiated in air and vacuum environments are systematically investigated. Raman spectroscopy indicates that there is no significant change in structural conformation of PEDOT:PSS film after gamma-ray irradiation. However, the conductivity of the film decreases as a function of dose in both air and vacuum environments, which can be deduced as a result of defects created in the structure. Hall effect measurements showed higher carrier concentration when the samples are irradiated under vacuum in comparison to the air environment, whereas mobility decreases as a function of dose irrespective of the environment. Furthermore, the electron spin resonance spectra provided evidence of the evolution of polaron population after gamma-ray exposure of 3 kGy, due to the decrease in charge delocalization and molecular ordering of the molecules. This decrease in conductivity and mobility of the PEDOT:PSS films irradiated in air and vacuum environments can be mainly ascribed to the defects and radical formation after gamma-ray exposure, favoring chain scission or cross-linking of the polymers.

## Abstract 2

Effects of gamma-ray irradiation (up to 3 kGy) on the structural and electronic properties of poly(3,4-ethylenedioxythiophene):poly(styrenesulfonate) (PEDOT:PSS), irradiated in air and vacuum environments are systematically investigated.

## Graphical abstract

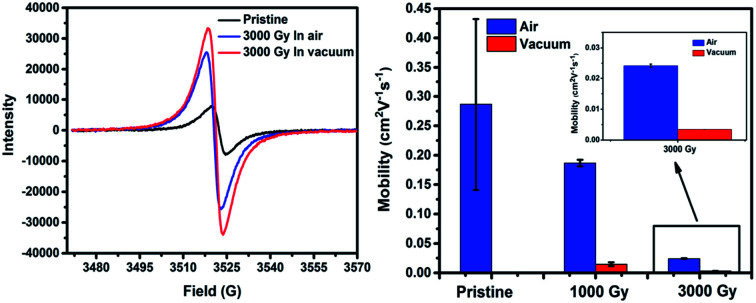

## 1. Introduction

Nowadays, the requirements for electrical components with higher conductivity, flexibility, and transparency of organic devices are increasing drastically. Among various conducting polymers available, poly(3,4-ethylenedioxythiophene) (PEDOT):polystyrenesulfonate (PSS) is a promising conducting polymer owing to its high conductivity, high solubility in water, and high stability in the oxidized state, favoring it to be utilized as a hole transport layer in organic light emitting diodes (OLEDs), backlights for liquid crystal displays, flexible touch screens and organic photovoltaic (OPV) cells.^1–9^ PEDOT:PSS can achieve a higher doping state by controlling its molecular structure arrangement and also by utilizing chemical treatment.^10–12^ In particular, it is essential to understand the ionizing effects of PEDOT:PSS films after exposure to high radiation environments for utilizing them in space and nuclear related applications. Therefore, the studies related to ionizing radiation effects based on PEDOT:PSS thin films are of keen interest.

High energy ionizing radiation such as proton,^13^ gamma-ray,^14–20^ UV treatment,^21–25^ and X-ray^15^ has been utilized as an alternative approach for tailoring the structural and electrical conductivities of the films. Usually, these approaches are temperature independent and in turn results in material with higher purity.^26^ The radical formation or displacement of atoms takes place after being exposed to ionizing radiation, which in turn results in the change of conductivity of the polymer films. Since, pristine PEDOT:PSS film contains both benzoid and quinoid conformation, structural correlation of these conformation tailoring the conductivity of the film needs to be verified. Previous reports confirmed that UV exposure on PEDOT:PSS films resulted in a red shift of the Raman spectra favoring quinoid conformation of PEDOT chain.^21,23,27^ Despite the structural changes caused by UV treatment, these samples also revealed increased conductivity, which in turn lead to the enhanced performance of the OPVs or OLED based devices.^21,23,24,27^ Therefore, in this work, resistivity measurements together with Raman spectroscopy were utilized to understand the structural conformation exists in PEDOT:PSS films. However, it is evident that not all kind of radiation treatment of PEDOT:PSS thin films lead to a decrease in resistivity. A trend of decreasing conductivity, together with the results obtained from Raman after gamma-ray exposure is contradictory with UV-irradiated samples as shown in Table 1. Therefore, it is interesting to scrutinize the mechanism of conductivity difference between gamma ray and UV ray irradiation. It is proposed that structural conformation in PEDOT after gamma-ray exposure, can be associated due to its higher penetration ability compared to UV, resulting in lesser radical formation in the PEDOT structure. In addition, the radical formations during irradiation are very active. Most importantly, the environment during irradiation (*i.e.*) either vacuum or air environment could result in a significant change in the structure, especially on the ultra-thin film materials. Since, the samples irradiated in air environment could promote cross-linking with oxygen resulting in fewer radicals and charge carriers, whereas radicals and charge carriers created may be higher for samples irradiated in vacuum environment.

**Table 1:** Comparison table of Raman spectra after exposure to various ionizing radiations

Type of irradiation	Raman scattering peak shift of thiophene rings	Raman scattering intensity change of ethylenedioxy ring peak	Conductivity	Ref.
UV	Red shift	Not available	Increase	Lin *et al.*,^27^
UV	Red shift	Not available	Increase	Lee *et al.*,^21^
UV	Red shift	Intensity drop	Increase	Tang *et al.*,^23^
Beta & gamma-ray	No shift but intensity degradation	Not available	Increase & decrease	Kane *et al.*,^16^
X-ray & gamma-ray	Blue shift	Not available	Decrease	Schrote *et al.*,^15^
Proton	Red shift	Not available	Increase	Singhal *et al.*,^13^
Gamma-ray together with ethylene glycol & ethylene diamine treatment	No shift	Not available	Decrease	Jang *et al.*,^14^
Gamma-ray irradiation in air and vacuum environment	No shift	Intensity drop slightly	Decrease	This work*

In this work, we focused on change in the structural conformation and electronic structure of PEDOT:PSS film after gamma-ray irradiation with respect to, air and vacuum environment. To understand the changes in the structural confirmation together with the electronic structure on spin coated PEDOT:PSS films before and after gamma-ray exposure, Raman spectroscopy and near edge X-ray absorption spectroscopy (NEXAFS) were studied. Furthermore, electron spin resonance (ESR) spectroscopy was employed to elucidate the free radicals formation such as polarons or bipolarons in the structure of PEDOT:PSS molecule after gamma-ray exposure. Finally, the changes in electrical properties such as resistivity, carrier concentration, and mobility have been studied to confirm the structural conformational change due to the ionizing radiation exposure in air and vacuum environments.

## 2. Experimental

PEDOT:PSS aqueous solution was purchased from Uni-onward Corporation (UR-AI4083) and spin coated at the rotation speed of about 1500 rpm on silicon wafers of 1 cm × 1 cm sized with a thermally grown oxide layer of 300 nm. The deposited samples were then dried at 110 °C in a hot plate for 10 min in the ambient atmosphere to remove the residual solvent. The thickness of the films were measured using ellipsometry technique.

Gamma-ray irradiation experiments were performed at Radioisotope Laboratory Facility of National Tsing Hua University by using ^60^Co source (29 kCi), whose energies are 1.173 MeV and 1.332 MeV. The as-prepared samples were separately irradiated at a dose rate of 1 kGy h^−1^ to achieve a total dose of up to 3000 Gy by varying the exposure time of the samples. In addition, the irradiation was carried out in air and vacuum environments to study the influence or role of free radicals formed under different irradiation environments. To irradiate the samples in vacuum, the as-deposited samples were sealed off to avoid their interaction with atmospheric oxygen.

Raman spectroscopy was performed at room temperature using a 532 nm excitation laser (HORIBA HR800). The crystal structure of PEDOT:PSS films were characterized by using Panalytical X'Pert Pro at Cu K_α_ (*λ* = 1.5406 Å). Atomic force microscopy (AFM) was used to determine the surface morphology and roughness of thin films before and after of gamma irradiation. The optical properties of PEDOT:PSS films before and after irradiation in air and vacuum environment was studied using photoluminescence (PL) spectrometer (Hitachi F-7000 Spectrometer) with an excitation wavelength of 540 nm. Electronic structure change of PEDOT:PSS films before and after irradiation were studied by utilizing NEXAFS studies (total electron yield mode) on C K-edges at beamline BL-20A in National Synchrotron Radiation Research Center (NSRRC), Taiwan. Variations in the chemical states of the films before and after irradiation at different irradiation environments were carried out using X-ray photoelectron spectroscopy (XPS) at the BL-24A beamline of NSRRC facility. XPS was performed at a photon energy of about 1150 eV. For the calibration purpose, Au standard was used after each sample. Besides, XPS spectra did not reveal the presence of other impurities. Electrical measurements were performed with the use of four-probe method (Keithley 2410 current source) and the probes were made of gold coated beryllium copper mixture with a diameter of about 100 μm. Mobility and carrier concentration of the films were characterized using the Hall-effect system ECOPIA-HMS 3000 and three measurements were carried out for each sample. Finally, ESR spectroscopy was adapted to characterize the nature of the charge carrier responsible for the change in conductivity of the samples. ESR spectra of the samples were collected using a Bruker EMX spectrometer at a microwave frequency of 9.861 GHz.

## 3. Results and discussions

**Fig. 1:**
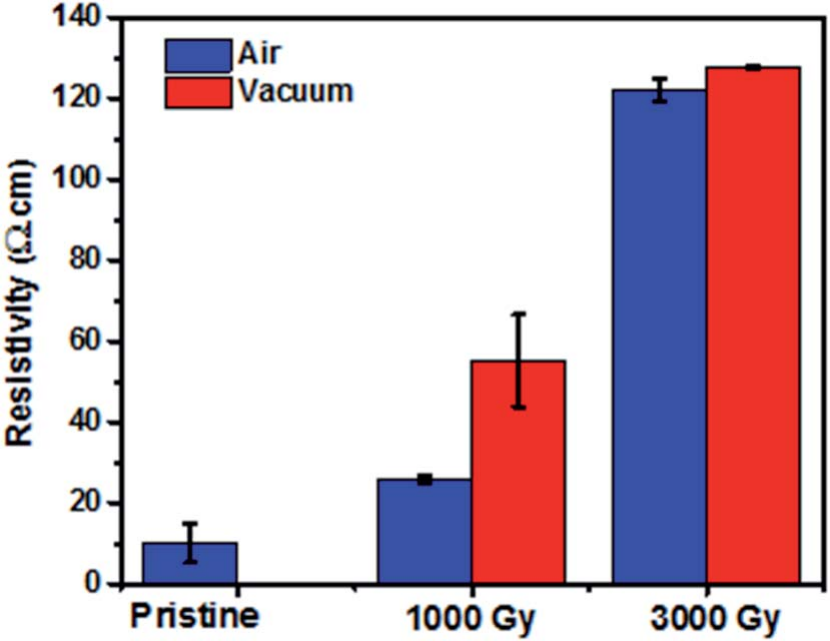
Change in resistivity as a function of dose for the samples irradiated in air and vacuum environment.

Fig. 1 shows the resistivity change as a function of dose at different irradiation environments. It can be noted that the resistivity increases as a function of dose, which is consistent with the work reported by Jang *et al.* and Kane *et al.*^14,16^ This phenomenon of decrease in conductivity of the samples at different doses irrespective of the irradiation environments could have resulted due to the defect formation and cross-linking of the polymers. Additional resistivity data of PEDOT:PSS films after irradiation at 500 Gy and 1500 Gy in air and vacuum environments has been shown in Fig. S1.†

**Fig. 2:**
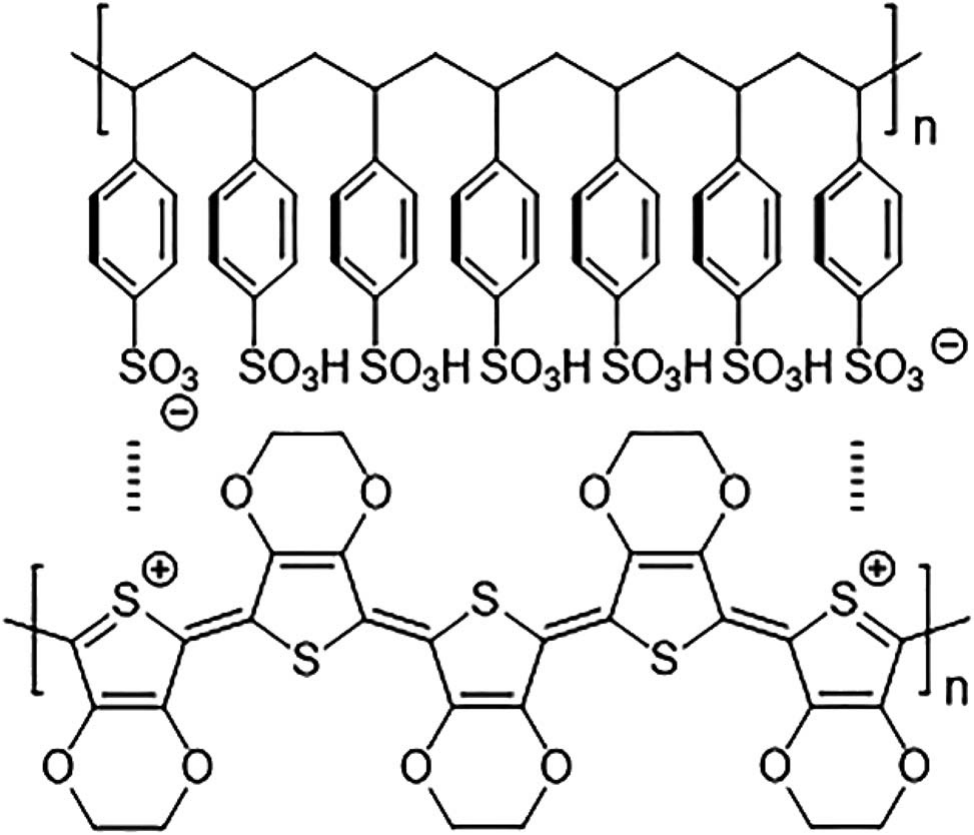
Chemical structure of PEDOT:PSS films reproduced from Gueye *et al.*^41^ Copyright, 2020 Elsevier Ltd.

**Fig. 3:**
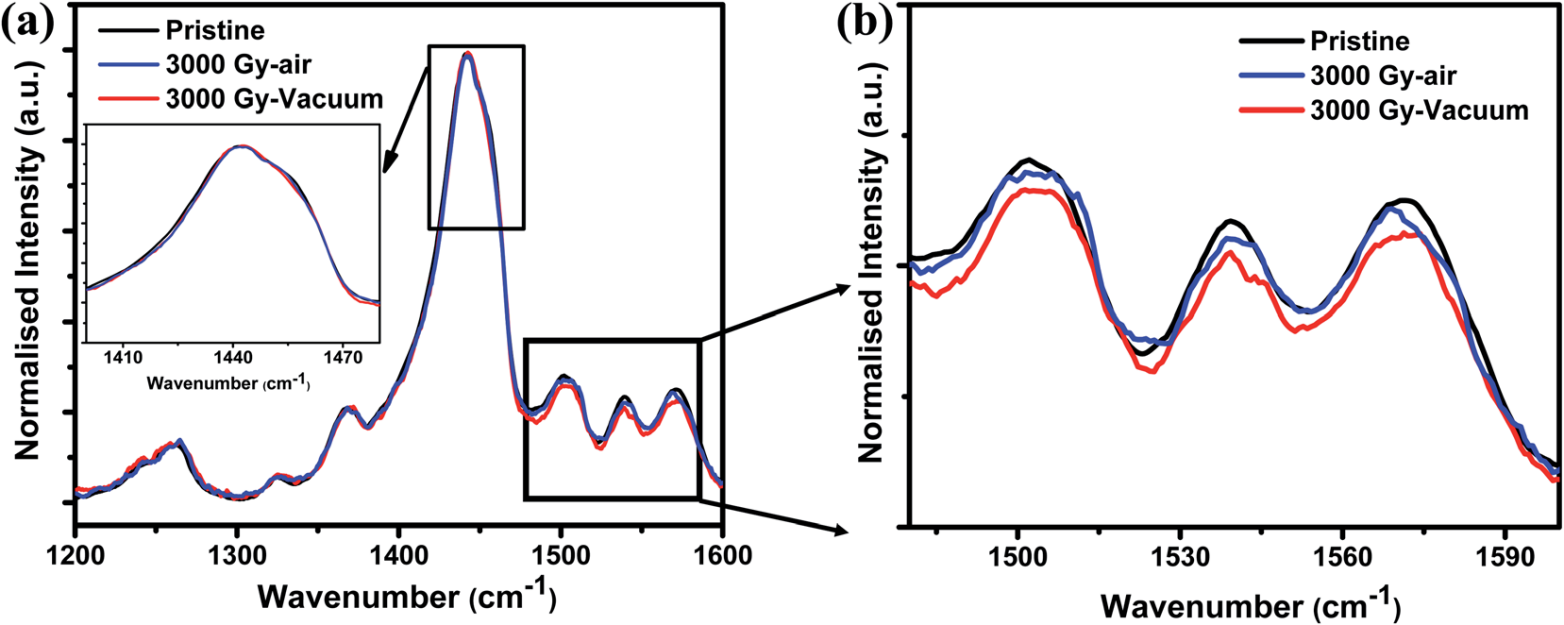
Raman spectrum of (a) pristine and 3 kGy irradiated PEDOT:PSS films at air and vacuum environment and (b) the enlarged view of ethylenedioxy ring.

The chemical structure of PEDOT:PSS films is shown in Fig. 2. To identify any conformational change in the structure of PEDOT:PSS films after gamma-ray irradiation, Raman spectroscopy was carried out. As shown in Fig. 3(a), the major characteristic features in PEDOT:PSS films are observed in the range of 1200–1600 cm^−1^. The peaks at 1265, 1444, and 1506 cm^−1^ are attributed to C_α_–C_α_′ inter-ring stretching vibration, C_α_=C_β_ symmetric stretching vibration on the five membered thiophene rings, and the out-of-plane bending of the ethylenedioxy ring of PEDOT chains, respectively.^28,29^ The peak at 1538 cm^−1^ arises from the splitting of asymmetric vibrations and peak at 1571 cm^−1^ corresponds to C_α_=C_β_ asymmetric stretching vibration.^30,31^ In general, pristine PEDOT:PSS films usually contains both benzoid and quinoid structural conformation.^23,32^ In a benzoid (coiled) conformation, the C_α_–C_β_ between two thiophene rings in the PEDOT chain is similar to σ bond, which usually has a low density of conjugated π-electrons. Whereas in the case of quinoid (linear) conformation, adjacent thiophene rings nearly lie on the same plane resulting in the delocalization of conjugated π-electrons throughout the PEDOT chain.^23,32^ Therefore, if the dominant conformation exists in PEDOT films is in the form of quinoid (linear) structure will lead to increased conjugated length of PEDOT chain, thereby resulting in enhanced conductivity of the sample. In case of thiophene rings, UV irradiation leads to the red shift of the peak as shown in Fig. 3(a), however, no such significant peak shift can be seen after gamma-ray exposure of the films irradiated in air and vacuum environments. This might be due to the amount of defects created in the polymer structure occupied only a small amount out of the whole volume. If the dose of gamma irradiation could be further increased by a few order of magnitude, the intensity drop of Raman can be observed as reported by Kane *et al.*^16^ Researchers reported other ionizing radiation induced experiments on PEDOT:PSS polymers such as using UV, electron or ion beam, and observed change in Raman spectra due to the created defects, which is reasonable (see Table 1). Whereas we used gamma ray, which has a very high penetration power and can penetrate through the entire thin film, thus the defect density is too low to be observed. Yet, there has been a decrease in the intensity of the peaks around 1500 cm^−1^, which is attributed to the ethylenedioxy ring of the PEDOT chain as shown in Fig. 3(b). Table 1 shows the comparison of Raman spectroscopy and conductivity change in PEDOT:PSS films after exposure to different ionizing radiations and chemical treatment. Maharajan *et al.* observed no peak shift in Raman spectra after thermal treatment of PEDOT:PSS films and suggested that thermal treatment only leads to conversion of PEDOT:PSS films into a glassy state. This thermal energy is not sufficient to cause any significant change in structural conformation of Raman spectra, but is reflected with feeble changes in chain alignment due to chain relaxation, which leads to the decrease in the intensities of asymmetric peaks.^33^ Based on these results, we speculate that gamma-ray irradiation could have favored only re-orientation of the polymer chain rather, than in the case of UV-irradiated samples, which cause a peak shift of about 15–20 cm^−1^. Even though, no noticeable peak shift can be observed, yet, it should be noteworthy that there is a possibility of reordering of chemical bonds after gamma-ray exposure. Because even a slight change in the structure will significantly affect the electrical properties for the various applications. Another possible reason for no such change in structural conformation might be due to the occurrence of chain scission or cross-linking in the polymer chain after exposure to gamma-ray irradiation,^34,35^ which will be discussed later.

All the X-ray diffraction data show no peak as shown in Fig. S2,† which confirmed the amorphous nature of the films before and after irradiation. This result is consistent with the work reported by Wang *et al.*^36^ Furthermore, AFM results confirmed the uniform growth of samples throughout the substrate. No noticeable change in the surface morphology can be observed before and after irradiation in air and vacuum environments up to 3 kGy as shown in Fig. S3.† And the thickness of the spin coated films lies around 60–70 nm.

**Fig. 4:**
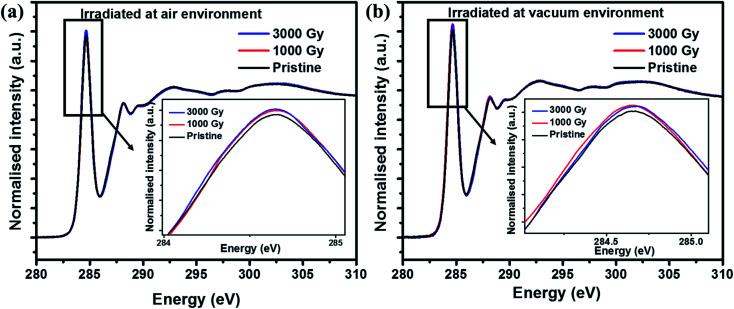
C1s NEXAFS spectra of PEDOT:PSS films irradiated at various doses in (a) air and (b) vacuum environment.

In addition, to characterize the changes in the electronic structure of the PEDOT:PSS films after irradiating in air and vacuum environments, NEXAFS studies were carried out. From Fig. 4, C1s NEXAFS spectra reveals the existence of different types of resonances. The peak around 284.5 eV corresponds to the π* transition from the aromatic carbon and is broadened due to the C–S bond. Whereas peak around 287 eV is related to C–H bonds associated with the alkane chain, together with the possible contributions from C–O bonding and the peak at 288.2 eV corresponds to the π* transition from C–H bonds associated with an aromatic ring. And finally transitions >290 eV corresponds to σ* transitions.^37,38^Fig. 4(a) shows the C1s NEXAFS spectra of PEDOT:PSS films irradiated in air environment and the results showed higher unoccupied π* states for the samples irradiated at 3 kGy. This result suggests the presence of a relatively higher amount of unoccupied density of states for irradiated samples in comparison to the pristine sample, whereas the attenuated intensity in case of pristine film is due to completely filled outermost orbital. Also, it can be seen from Fig. 4(b) that the C1s spectra follow a similar trend for the samples irradiated in the vacuum environment, where 3 kGy irradiated sample has slightly more unoccupied π* states in comparison with the pristine sample. Altogether from C1s NEXAFS spectra reveals that there is an increased unoccupied π* states after irradiation due to the defects or radicals created along the long chain of the PEDOT molecule irrespective of air and vacuum environments.

**Fig. 5:**
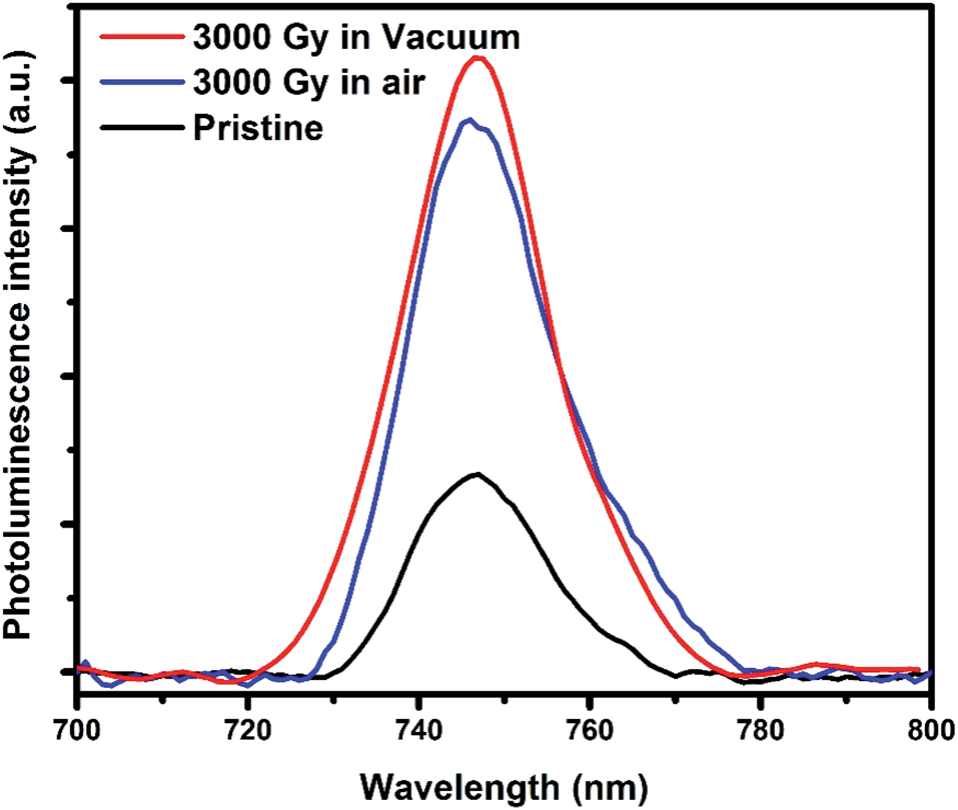
PL spectra of pristine and irradiated PEDOT:PSS films in air and vacuum environment.

To interpret the defects created in the PEDOT:PSS films before and after irradiation, PL studies has been carried out. From Fig. 5, the PL intensity of pristine PEDOT:PSS film is smaller in comparison to the gamma-irradiated samples in air and vacuum environments. In other words, it means that the density of defect states in irradiated samples are larger than the pristine one. This result is consistent with the resistivity measurement and ESR spectroscopy results, which also revealed the better conductivity and presence of less defects in pristine as compared to the irradiated samples.

**Fig. 6:**
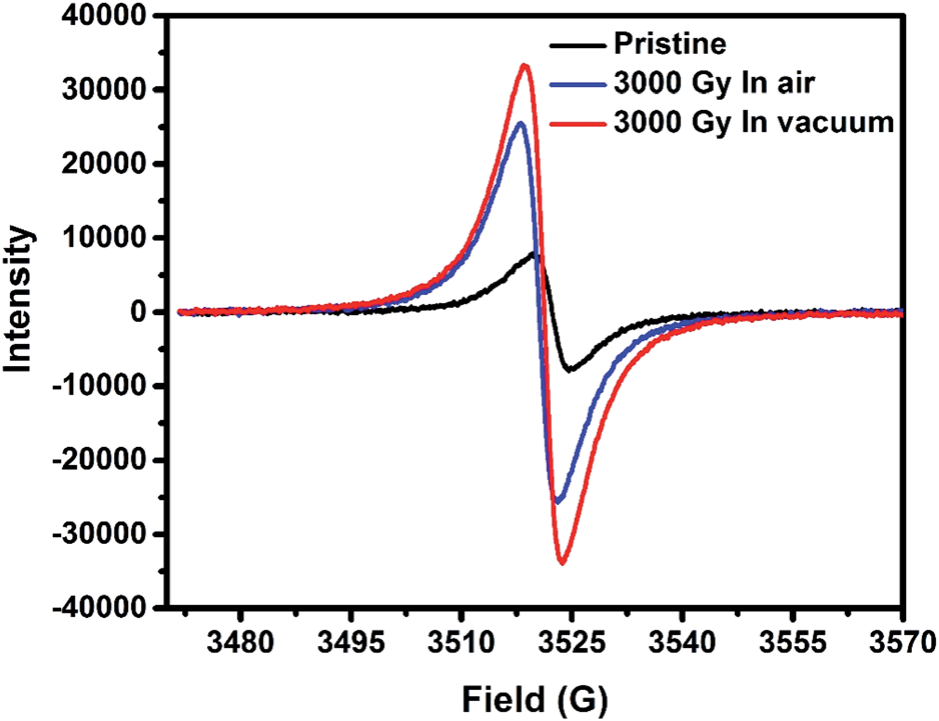
ESR spectra of pristine and irradiated PEDOT:PSS films exposed in different irradiation environment.

ESR spectroscopy technique of PEDOT:PSS films revealed a *g*-factor of about 2.0 for all the samples, which is typically due to the presence of free non-spin-paired electrons delocalized across the conjugated π-system.^39^ From Fig. 6, it can be seen that the pristine PEDOT:PSS samples revealed a broad spectrum in comparison to irradiated samples, which is attributed to a higher degree of charge delocalization across the conjugated PEDOT backbone of the molecule. Whereas irradiation in air and vacuum environments leads to the evolution of bipolaron (*s* = 0) to the polaron (*s* = 1/2) population resulting in the decreased conductivity of the films. Polarons possess a spin of 1/2 which could be detected by ESR, whereas bipolarons have integer spin that could be not be detected in ESR spectra.^40^ Therefore, we attribute the increase in ESR spectroscopy intensity after irradiation is due to the transformation of the nature of charge carriers from bipolarons to polarons. This leads to a decrease in charge delocalization as well as the molecular orientation of the PEDOT molecule since bipolarons are more favorable for charge transportation than polarons along the polymer backbone.^15^ The linewidth (deltaHpp) of the pristine PEDOT:PSS films lies around 5.3 G, whereas the linewidth of the gamma-irradiated PEDOT:PSS films are around 4.8 G. Thus, higher degree of charge delocalization is favorable for better conductivity in pristine samples in comparison to the irradiated films in air and vacuum environments.^32^

**Fig. 7:**
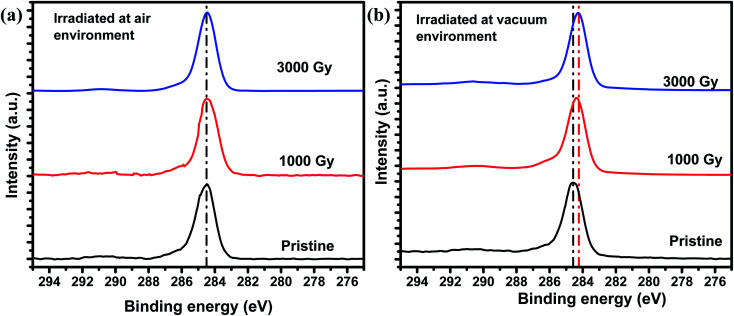
C1s XPS spectra of PEDOT:PSS films irradiated at various doses in (a) air and (b) vacuum environment.

Fig. 7(a) and (b) shows the XPS spectra of the C1s peak before and after irradiation in air and vacuum environments. XPS spectra revealed a slight binding energy (B.E.) shift for samples irradiated in air environment, which seems to be under the statistical limits. However, in the case of samples irradiated in vacuum environment the B.E. shifts to lower energy by around 0.3 eV. This shift towards lower B.E. could be attributed to the radical formations resulting in the conversion of a population of bipolarons to polarons, which is consistent with our ESR spectra. Whereas no significant B.E. shift in case of samples irradiated at air environment because there is a chance that the radicals formed reacts with oxygen in the air. This B.E. shift in vacuum environment might have occurred due to the lack of radicals recombine with oxygen in the atmosphere in comparison to the samples irradiated in air environment. Meanwhile, these results also seem consistent with Fig. 3, where the Raman scattering intensity corresponding to ethylenedioxy ring decreased slightly in air in comparison to the sample irradiated in a vacuum environment.

**Fig. 8:**
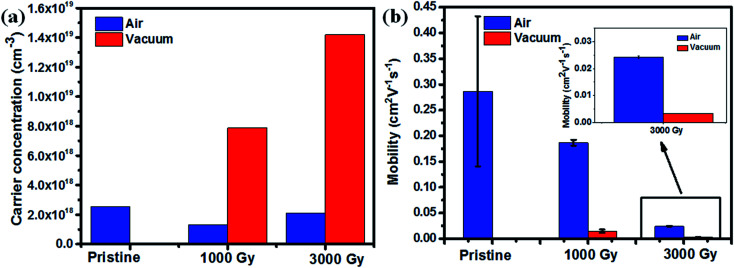
(a) Carrier concentration as a function of dose, (b) mobility change as a function of dose at different irradiation environment and the inset shows the enlarged view of mobility of 3000 Gy.

Finally, to correlate the results obtained from ESR, Raman spectroscopy and resistivity, carrier concentration and mobilities of the PEDOT:PSS films, before and after gamma-ray irradiation, under different environments were measured by using Hall measurement with van der Pauw method. The carrier mobility of the samples were calculated using *σ* = *enμ*, where *σ* is conductivity, *e* is the unit charge, *n* is carrier concentration, and *μ* is carrier mobility. The earlier discussed NEXAFS data revealed that the unoccupied density of states of the PEDOT:PSS molecules have been enhanced after gamma-ray irradiation, which indicates the possibility of the formation of intermediate states created after gamma-ray exposure. This in turn may have resulted in boosting up the carrier transport of the molecule, resulting in an increase in carrier concentration as shown in Fig. 8(a), owing to the radicals or defects created in the molecule. It can be also noted that the number of charge carriers available is higher for samples irradiated in a vacuum environment in comparison to the samples irradiated in air environment. However, for 1 kGy sample irradiated in air environment, the carrier concentration gets reduced, which might be due to the oxygen in air favoring cross-linking of polymers at a lower dose. As the dose level increases to 3 kGy or higher, chain scission eventually dominates resulting in a higher carrier concentration. These results are also consistent with the ESR spectroscopy results, which revealed the number of free carriers or radicals are higher in case of samples irradiated in a vacuum environment to the sample irradiated in air environment. Also, it can be seen from Fig. 8(b) that the mobility gets reduced as a function of dose, which may be due to the chain breaking of the conducting polymer. It can be noted further that the mobility of vacuum irradiated samples becomes poorer than the samples irradiated in air environment. This could have been possible along the PEDOT molecular chain because coiled like structural conformation with more disordered structure leads to a higher chance of hole scattering, thereby reducing the mobility of the films irradiated in a vacuum environment more steeply in comparison to the samples irradiated in an air environment.

Chain scission and cross-linking of the polymers are the two plausible mechanisms that can explain the increase in resistivity of the samples as shown in Fig. 1. Generally, when polymer films are irradiated by ionizing radiation, free radicals are usually formed along the molecular chain of the PEDOT molecule. Cross-linking is attributed to the recombination of free radicals, whereas chain scission favors the formations of free radicals along the polymer chain. In case of PEDOT:PSS films irradiated in air, the initial decrease in conductivity is attributed to the dominant mechanism favoring cross-linking of the polymers leading to the reduction in the carrier concentration of the samples. Whereas at higher dose, free radical formations dominate further resulting in the decrease of the conductivity of the samples. Although the carrier concentrations are all increased for the sample irradiated in air and vacuum at 3 kGy, the chemical bonds broken due to gamma-ray exposure reduce the conjugation length resulting in much lower mobility and finally gives rise to lower conductivity.

In overall, this study revealed that gamma-ray irradiation in air and vacuum environments has different behaviors towards the carriers responsible for electron transport. Furthermore, this study suggests that the mechanism in gamma-ray irradiated films observed is different from the conductivity enhancement obtained after UV irradiation.

## 4. Conclusion

In this study, spectroscopy and post-irradiation analysis were utilized to interpret the changes in the structural conformation and electronic properties of PEDOT:PSS films after gamma-ray exposure in air and vacuum environments. Raman spectroscopy revealed no significant change in the structural conformation after exposure to gamma-ray irradiation of up to 3 kGy in air and vacuum environments. Meanwhile, electrical properties such as conductivity and mobility steeply drop as a function of dose in both air and vacuum environments. The Hall-effect measurements revealed higher carrier concentration for samples irradiated in vacuum environment in comparison to the samples irradiated in air environment. These results can be correlated with ESR spectroscopy results, which further confirmed the evolution of population of the non-spin-paired polaron (*s* = 1/2) upon exposure to gamma-ray irradiation due to the decrease in charge delocalization as well as the molecular disorders along with the π–π stacking of the conjugated polymer chain. Overall, this work suggests that defects together with charge carriers created after gamma-ray exposure might have favored chain scission or cross-linking of the polymers resulting in the decrease of conductivity and mobility of the PEDOT:PSS films exposed at different irradiation environments.

## Conflicts of interest

The authors declare no conflict of interest.

## Supplementary Material

RA-011-D1RA03463D-s001
