# Correction: Consequences of gamma-ray irradiation on structural and electronic properties of PEDOT:PSS polymer in air and vacuum environments

**DOI:** 10.1039/d1ra90130c

**Published:** 2021-07-01

**Authors:** Aswin kumar Anbalagan, Shivam Gupta, Mayur Chaudhary, Rishi Ranjan Kumar, Yu-Lun Chueh, Nyan-Hwa Tai, Chih-Hao Lee

**Affiliations:** Department of Engineering and System Science, National Tsing Hua University Hsinchu 30013 Taiwan chlee@mx.nthu.edu.tw; Department of Materials Science and Engineering, National Tsing Hua University Hsinchu 30013 Taiwan; Institute of Nuclear Engineering and Science, National Tsing Hua University Hsinchu 30013 Taiwan

## Abstract

Correction for ‘Consequences of gamma-ray irradiation on structural and electronic properties of PEDOT:PSS polymer in air and vacuum environments’ by Aswin kumar Anbalagan *et al.*, *RSC Adv.*, 2021, **11**, 20752–20759, DOI: 10.1039/D1RA03463D.

The authors regret that incorrect details were given for ref. 18. The correct version of ref. 18 is given here as ref. 1.

The Royal Society of Chemistry apologises for these errors and any consequent inconvenience to authors and readers.

## Supplementary Material
