# Supplementary material for: Consequences of gamma-ray irradiation on structural and electronic properties of PEDOT:PSS polymer in air and vacuum environments
Source: RSC Adv. 2021 Jun 10;11(34):20752–9. doi: 10.1039/d1ra03463d (PMC9034154; doi:10.1039/d1ra03463d)
Supplement: RA-011-D1RA03463D-s001 [file RA-011-D1RA03463D-s001.pdf]

Electronic Supplementary Material (ESI) for RSC Advances.
This journal is © The Royal Society of Chemistry 2021

Supplementary information

For

Consequences of gamma-ray irradiation on structural and electronic properties of PEDOT: PSS polymer under air and vacuum environments

Aswin kumar Anbalagan [a], Shivam Gupta [b], Mayur Chaudhary [b], Rishi Ranjan Kumar [b], Yu-Lun Chueh [b], Nyan-Hwa Tai [b] and Chih-Hao Lee [a,c,*]

[a] Department of Engineering and System Science, National Tsing Hua University, Hsinchu, Taiwan, 30013

[b] Department of Materials Science and Engineering, National Tsing Hua University, Hsinchu, Taiwan, 30013

[c] Institute of Nuclear Engineering and Science, National Tsing Hua University, Hsinchu, Taiwan, 30013

[*] Corresponding author: chlee@mx.nthu.edu.tw

Resistivity measurement

Figure S1 shows the resistivity change as a function of dose for PEDOT: PSS films irradiated in air and vacuum environment at 0, 500, 1000, 1500 and 3000 Gy, respectively.

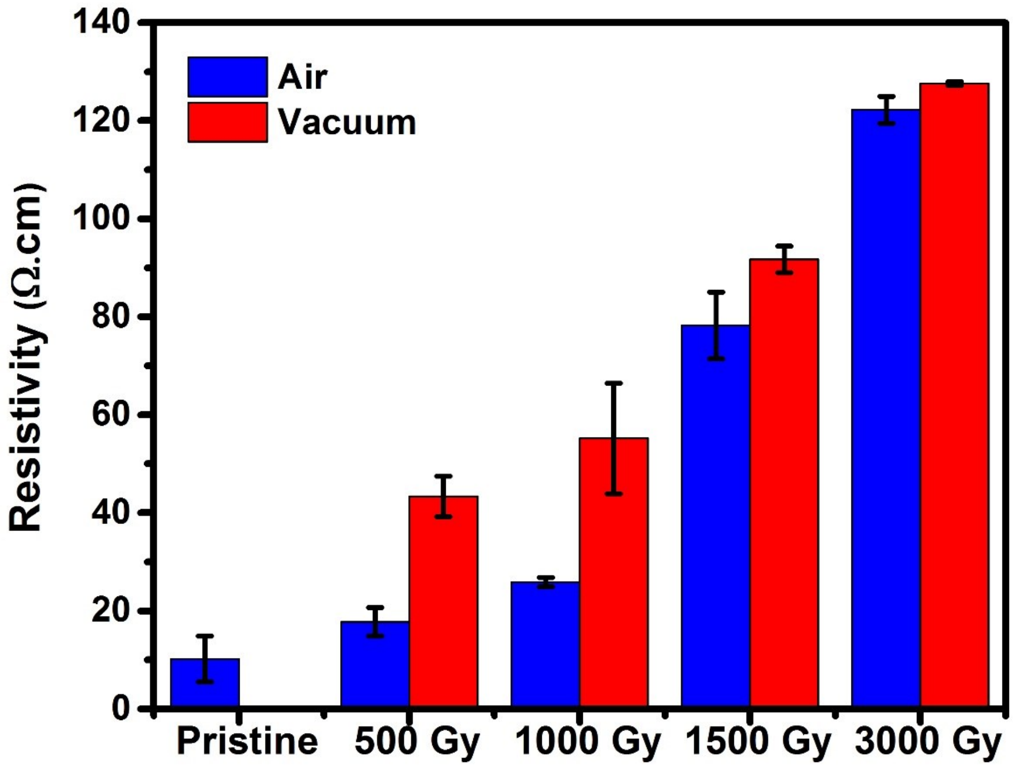

Figure S1. Resistivity change as a function of dose for the PEDOT: PSS films irradiated in air and vacuum environment.

X-ray diffraction (XRD)

XRD studies revealed the amorphous nature of PEDOT: PSS films before and after irradiation in air and vacuum environment up to 3 kGy as shown in **Figure S2**.

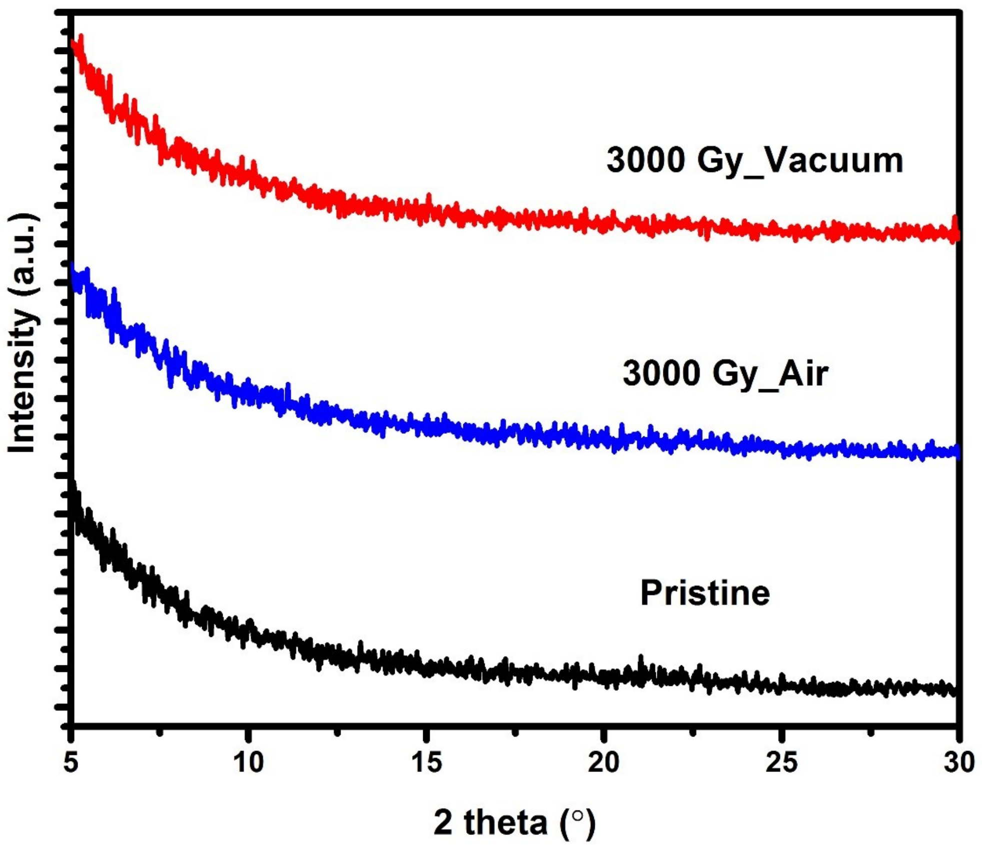

Figure S2. XRD of pristine and 3000 Gy gamma-irradiated PEDOT: PSS films in air and vacuum environment.

Atomic Force Microscopy (AFM)

AFM was performed by tapping mode across 10 μm * 10 μm at various positions for pristine and irradiated samples at air and vacuum environments. **Figure S3** revealed no significant difference in the morphology of the samples before and after irradiation.

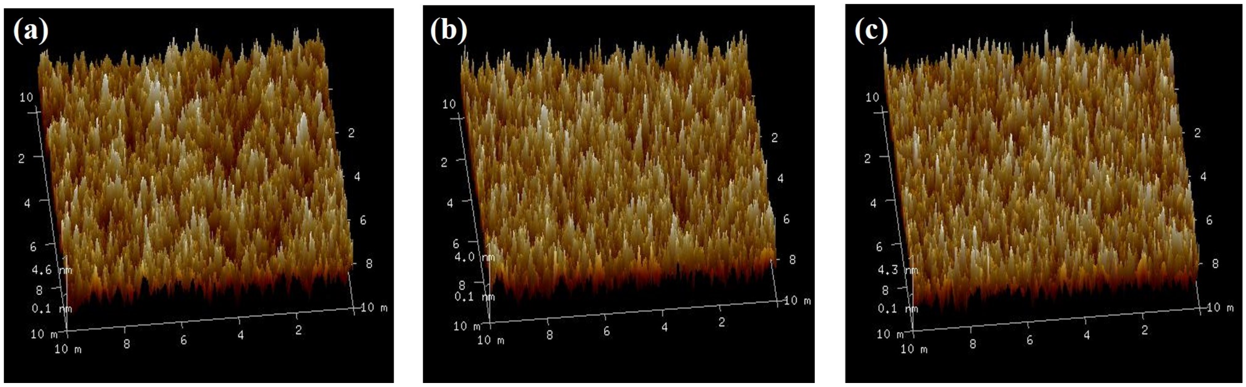

Figure S3. AFM topographies (10 μm x 10 μm) of: (a) Pristine and 3000 Gy irradiated PEDOT: PSS films: (b) in air and (c) vacuum environment.
